# RhoA Controls Wnt Upregulation on Microstructured Titanium Surfaces

**DOI:** 10.1155/2014/401859

**Published:** 2014-05-14

**Authors:** Simone Lumetti, Silvia Mazzotta, Sara Ferrillo, Maddalena Piergianni, Marilina Piemontese, Giovanni Passeri, Guido Maria Macaluso, Carlo Galli

**Affiliations:** ^1^Section of Periodontology and Implant Dentistry, Centro di Odontoiatria, University of Parma, Via Gramsci 14, 43126 Parma, Italy; ^2^Department of Clinical and Experimental Medicine, University of Parma, Via Gramsci 14, 43126 Parma, Italy; ^3^Department of Biomedical, Biotechnological, and Translational Sciences, University of Parma, Via Gramsci 14, 43126 Parma, Italy

## Abstract

Rough topography enhances the activation of Wnt canonical signaling in vitro, and this mediates its effects on cell differentiation. However, the molecular mechanisms underlying topography-dependent control of Wnt signaling are still poorly understood. As the small GTPase RhoA controls cytoskeletal reorganization and actomyosin-induced tensional forces, we hypothesized that RhoA could affect the activation of Wnt signaling in cells on micropatterned titanium surfaces. G-LISA assay revealed that RhoA activation was higher in C2C12 cells on rough (SLA) surfaces under basal conditions than on smooth (Polished) titanium. Transfection with dominant negative RhoA decreased Wnt activation by normalized TCF-Luc activity on SLA, whilst transfection with constitutively active RhoA increased TCF-Luc activation on Polished titanium. One mM Myosin II inhibitor Blebbistatin increased RhoA activation but decreased Wnt activation on SLA surfaces, indicating that tension-generating structures are required for canonical Wnt modulation on titanium surfaces. Actin inhibitor Cytochalasin markedly enhanced RhoA and TCF-Luc activation on both surfaces and increased the expression of differentiation markers in murine osteoblastic MC3T3 cells. Taken together, these data show that RhoA is upregulated in cells on rough surfaces and it affects the activation of Wnt canonical signaling through Myosin II modulation.

## 1. Introduction

Surface profile is one of the major determinants of cell and tissue responses to endosseous implants. A large amount of experimental evidence over the years has demonstrated that rough topography can affect the differentiation of mesenchymal and bone cells, bolstering the expression of osteoblast-specific genes [1–3]. Several biochemical mechanisms have been advocated, such as prostaglandin secretion and paracrine signaling, and consensus has been reached about optimal roughness to support the differentiation of bone cells, but little insight is available as to how cells can sense the geometrical features of the implant surface and transduce them into prodifferentiative stimuli. Kilian et al. have shown that cell shape on micropatterned substrates can alter cytoskeletal organization and has dramatic effects on the fate of mesenchymal cells to specific lineages [4]. More specifically, star-like shapes were shown to promote osteoblastic differentiation, in contrast to rounder morphologies associated with adipocytic commitment. The organization of the cytoskeleton is therefore central to the differentiation process, and, among the cellular pathways that control cell shape, the Wnt noncanonical planar cell polarity (PCP) pathway stands out as one of the most effective determinants of cytoskeletal arrangement. As the Wnt canonical signaling cascade has been shown to be affected by substrate microtexture [5–7], the present study investigated whether some of the main controllers of cytoskeletal organization, the small GTPase RhoA, which is central to the PCP pathway, and its downstream targets actin and Myosin II are involved in topography mediated Wnt upregulation and how they contribute to cells' ability to react to surface topography.

## 2. Materials and Methods

### 2.1. Titanium Surfaces

Polished and acid-etched, sand-blasted (SLA) commercially pure titanium samples were kindly provided by Straumann Institut AG (Basel, Switzerland). These surfaces have been extensively described and characterized in the literature [8–10]. The samples were provided as sterile discs of 1 mm thickness and 16 mm diameter and were used in 24 well plates (Euroclone, Pero, MI, Italy) for the biological assays.

### 2.2. Cell Cultures

The C2C12 cell line was obtained from the European Catalog of Cell Cultures (Health Protection Agency Culture Collections, Salisbury, UK) and was grown in Dulbecco modified MEM (DMEM, Paa, Pasching, Austria), 10% fetal bovine serum (FBS, Gibco, Life Technologies Italy, San Giuliano Milanese, MI, Italy), 1% penicillin and streptomycin (Penstrep, Sigma Aldrich, St. Louis, MO, USA), and 1% Glutamine (Sigma Aldrich). C2C12 cells are an uncommitted, promyoblastic murine cell line that displays the ability to differentiate along both the myocytic and osteoblastic lineages, in the presence of appropriate cues. Due to their abundant expression of Wnt molecular machinery, they are a well- established in vitro model to investigate the regulation of Wnt canonical signaling. The MC3T3-E1 cell line was obtained from the American Type Culture Collection (LGC Standards S.r.L., Sesto S. Giovanni, MI, Italy) and cultured in DMEM medium as described above. MC3T3 are osteoblastic cells from mouse calvaria. They typically retain strong similarities with primary cells, such as contact inhibition, and are thus an established in vitro model of osteoblasts. To perform the viability assay and for gene expression, 100000 C2C12 cells were plated on Polished or SLA discs in 1 mL of complete medium in 24 well plates (Corning, Tewksbury, MA, USA), in triplicate and assayed 24 hours after plating. For transfection and reporter assays, C2C12 cells were plated on titanium surfaces in 1 mL/well Opti-MEM (Life Technologies, Italy), 5% FBS, 1% Penstrep at the density of 120000 cells/well and the cells were assayed after 24 hours. For differentiation assay, 100000 MC3T3 cells were seeded on titanium surfaces in complete DMEM. Culture medium was replaced by fresh medium containing 250 mM ascorbic acid (Sigma-Aldrich, St. Louis, MO, USA) 24 hours after plating and cells were cultured for 3 days prior to RNA extraction.

### 2.3. Inhibitors

For Blebbistatin and Cytochalasin experiments, cells were plated as described above and stimulated with 0.2 mM Cytochalasin or 1 mM Blebbistatin (Inalco, Milano, Italy) four hours after plating for the whole duration of the experiments. An equal amount of PBS was used as a control. Pilot experiments were performed to choose the reagent concentrations, which were chosen based on the literature as the minimal dose to induce consistent and predictable morphological changes at immunofluorescence.

### 2.4. RhoA Measurement

RhoA activation was measured using luminescence-based G-LISA Activation kit (Cytoskeleton Inc., Denver, CO, USA) according to the manufacturer instructions. Values were normalized by protein content using a colorimetric assay (Bio-Rad Laboratories, Hercules, CA, USA) according to manufacturer's recommendations.

### 2.5. Constructs

The TCF-Luc assay kit was purchased from SABioscience (Frederick, MD, USA). The kits contained a vector carrying the* Firefly* Luciferase gene under the control of a TCF-binding regulatory element and a control plasmid carrying a constitutively expressed* Renilla* Luciferase gene under the control of the CMV promoter. Luciferase activity was calculated as the ratio between* Firefly* and* Renilla* Luciferase. The dominant negative RhoA (plasmid 12152: pTriEx-RhoA FLARE.sc Biosensor T19N) and constitutively active (plasmid 12151: pTriEx-RhoA FLARE.sc Biosensor Q63L) isoforms were obtained from public Addgene plasmid repository and were kindly shared by the Klaus Hahn Lab [11].

### 2.6. Reporter Assays

The reporter assays were performed with dual-Luciferase reporter assay system (Promega Italy, Milan) according to the manufacturer's recommendations. For background reading, recombinant mouse Wnt3a was used. The Wnt3a recombinant protein was obtained from R&D systems (Minneapolis, MN, USA).

The samples were read with a Glomax 20/20 Luminometer (Promega) with double injectors.

### 2.7. Real-Time PCR

Total RNA was purified from cell cultures using Trizol (Life Technologies Italy, San Giuliano Milanese, MI, Italy) according to the manufacturer's directions. TaqMan quantitative RT-PCR was performed as previously described using the following primer probe sets from Applied Biosystems (Foster City, CA, USA): Alkaline Phosphatase (Mm00475834_m1); Osteocalcin (for 5′-GCTGCGCTCTGTCTCTCTGA-3′; rev 5′-TGCTTGGACATGAAGGCTTTG-3′; probe 5′-FAM-AAGCCCAGCGGCC-NFQ-3′). Mouse ribosomal protein S2, ChoB (for 5′-CCCAGGATGGCGACGAT-3′; rev 5′-CCGAATGCTGTAATGGCGTAT-3′; probe, 5′-FAM-TCCAGAGCAGGATCC-NFQ-3′) was used as housekeeping gene.

### 2.8. Immunofluorescence

Cells were seeded at the concentration of 20000 cells/well in complete medium, treated with vehicle or the inhibitor as described above and after 48 hours they were fixed with 4% paraformaldehyde (Sigma-Aldrich, St.Louis, MO, USA) for 10 min followed by three rinses with PBS. They were then permeabilized with 0.1% Triton-X100 (Sigma-Aldrich, St. Louis, MO, USA) for 5 min followed by three rinses with PBS. Nonspecific binding sites were blocked by incubating the samples in 1% bovine serum albumin in PBS for 20 min. Staining was then performed on each sample with TRITC-conjugated phalloidin (FAK100, Chemicon, Billerica, MA, USA) for 1 hour, followed by three rinses with PBS. Nuclear counterstaining was performed by incubation with DAPI (D1306, Molecular Probes, Life Technologies) for 5 min followed by three rinses with PBS. All the steps were carried out inside the culture well at room temperature. The treated discs were then transferred to microscope slides and were mounted under glass cover slips using an antifade-mounting medium (P7481, Molecular Probes, Life Technologies) for photo bleaching reduction. Samples were examined using a Nikon Eclipse 90i (Nikon, Tokyo, Japan) microscope equipped for fluorescence analysis. Heat maps were generated with freeware ImageJ software (NIH, Bethesda, MD, USA) using the inbuilt plugin.

### 2.9. Statistical Analysis

Data were analyzed using Prism 4 (GraphPad, La Jolla, CA, USA). All values are reported as the mean ± Standard Deviation of three repeated experiments. Differences between group means were evaluated with two-way ANOVA statistical test and Bonferroni posttest and differences were considered significant when *P* < 0.05.

## 3. Results

### 3.1. Surface Topography Affects Activation Levels of RhoA

**Figure 1:**
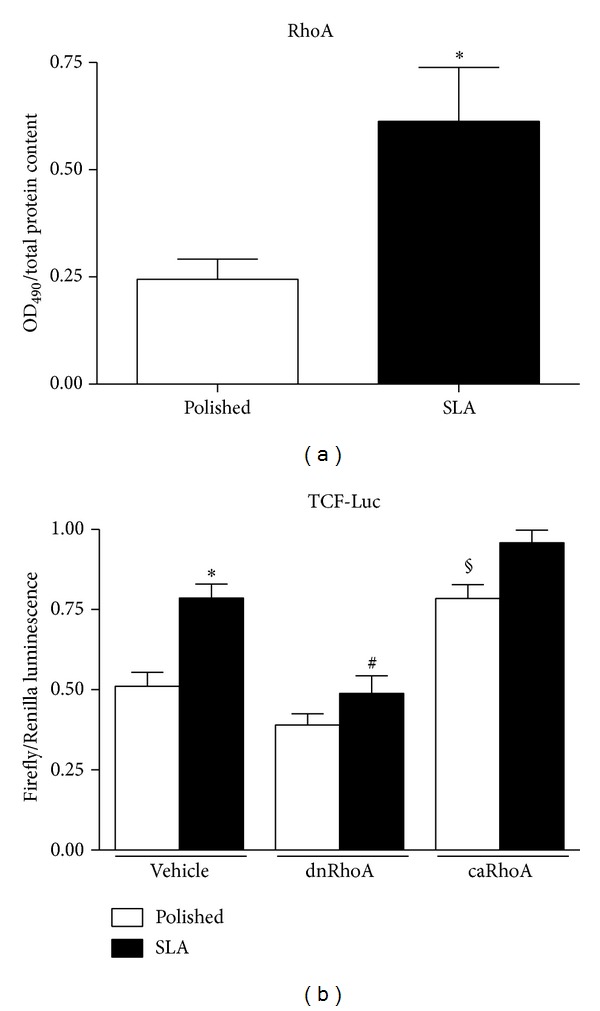
(a) Total RhoA activation levels in MC3T3 cells growing on Polished or SLA titanium surfaces by G-LISA assay. ^*^ *P* < 0.05 versus Polished. (b) Canonical WNT activity by Luciferase reporter assay in C2C12 cells on Polished or SLA surfaces after RhoA inhibition by dominant negative isoform (dnRhoA) or activation by transfection with constitutively active isoform (caRhoA). ^*^ *P* < 0.01 versus vehicle-treated Polished; ^#^ *P* < 0.01 versus vehicle-treated SLA; ^§^ *P* < 0.01 versus vehicle-treated Polished.

To investigate whether surface topography could affect the activation of the small GTPase RhoA, we measured RhoA activity in calvaria osteoblastic MC3T3 cells cultured on smooth (“Polished”) or rough (“SLA”) titanium discs by G-LISA assay, which quantitates the amount of GTP-loaded RhoA. The total amount of active RhoA in cells, normalized per protein content, was significantly (*P* < 0.05) higher in cells growing on rough surfaces after 24 hours of culture (Figure 1(a)).

### 3.2. RhoA Affects Activation of Canonical Wnt Signaling

Since RhoA is an important effector of the noncanonical Wnt planar cell polarity pathway, we sought to investigate whether this enzyme could affect the activation of canonical Wnt signaling in C2C12 cells growing on titanium surfaces. C2C12 cells are a premyoblast cell line and a popular model for Wnt studies because they possess abundant molecular machinery for this pathway. RhoA activation was modulated by transiently transfecting C2C12 cells on Polished or SLA surfaces with dominant negative or constitutively active RhoA isoforms [11] and by cotransfecting them with a reporter system including a plasmid vector carrying a Firefly Luciferase gene under the control of a TCF-responsive element and a constitutively active Renilla Luciferase gene for normalization. Background Wnt stimulation was provided by adding 1.5 mL/mL recombinant Wnt3a to the supernatant, as previously described [5]. As previously reported [5], Wnt activation was higher in cells growing on SLA surfaces (Figure 1(b), ^*^ *P* < 0.01). Inhibition of RhoA activity abolished canonical Wnt activation on SLA surfaces (^#^ *P* < 0.01 versus vehicle treated SLA), whilst constitutive activation of RhoA slightly but significantly increased the normalized TCF-Luc signal on Polished (*P* < 0.01). These results indicate that activation of RhoA may directly affect activation of canonical Wnt signaling in cells growing on micropatterned titanium surfaces.

### 3.3. Cytoskeletal Tension Modulates Cell Responses to Surface Topography

**Figure 2:**
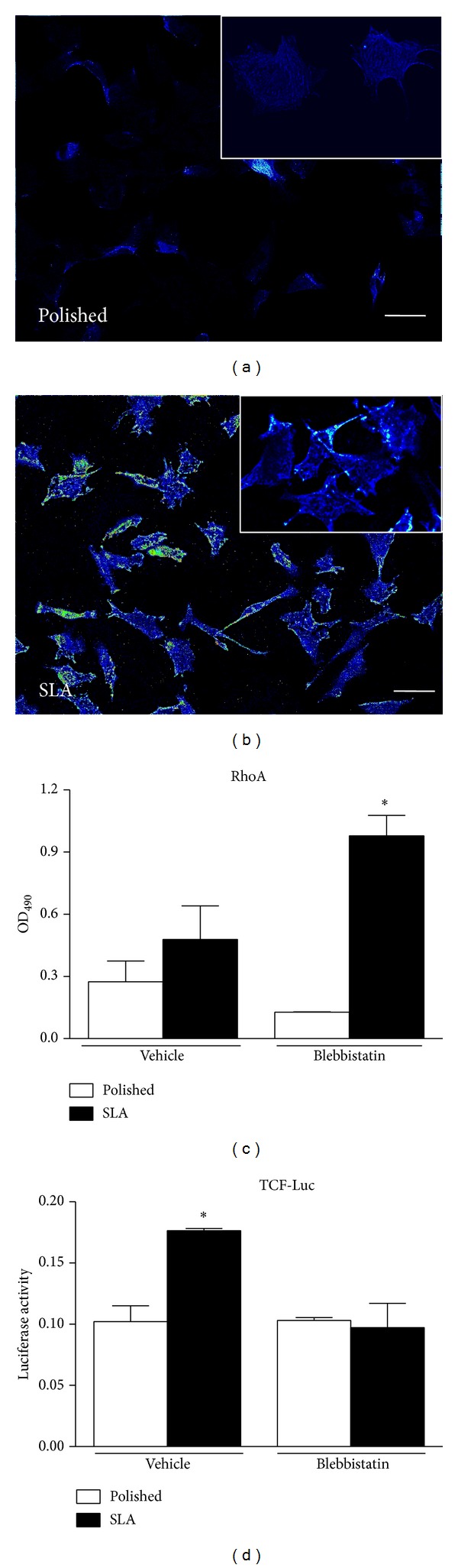
(a)-(b) Immunocytochemistry of Myosin II distribution in MC3T3 cells on Polished or SLA surfaces (20x magnification, bar 20 mm, insets: 40x magnification). Heat maps were generated using ImageJ freeware software. (c) Total RhoA activation levels in MC3T3 cells growing on Polished or SLA titanium surfaces by G-LISA assay after Myosin II inhibition by 1 mM Blebbistatin. ^*^ *P* < 0.01 versus vehicle-treated SLA, *P* < 0.001 versus Blebbistatin-treated Polished. (d) Canonical WNT activity by Luciferase reporter assay in C2C12 cells on Polished or SLA surfaces after Myosin II inhibition by 1 mM Blebbistatin. ^*^ *P* < 0.01 versus vehicle-treated Polished and Blebbistatin-treated SLA.

To better understand the relationship between cytoskeletal organization and Wnt activation, we visualized the distribution of Myosin II in MC3T3 cells on Polished or SLA surfaces (Figures 2(a) and 2(b)). Myosin II is a nonmuscle isoform of Myosin, widely expressed in cells of any phenotype, and is responsible for cell internal tension, also known as prestress levels in cell biomechanics [12]. Cells growing on smooth or rough titanium discs differed not only in their shape, as it is already well documented in the literature, but in Myosin distribution as well. Myosin label was stronger in cells on SLA surfaces after 48 hours of culture, especially along the cell edges, where they adapted to the underlying peaks and cavities (Figures 2(a) and 2(b)). Interestingly, inhibition of Myosin II activation by 1 mM Myosin Light Chain Kinase inhibitor Blebbistatin significantly increased RhoA activity in MC3T3 cells as measured by G-LISA assay (Figure 2(c), ^*^ *P* < 0.01 versus vehicle-treated SLA). As RhoA controls Myosin II activation via the Myosin Light Chain Kinase enzyme; this finding suggests a possible feedback loop control from Myosin or a tension-sensitive structure back to RhoA. However, Myosin inhibition by Blebbistatin decreased activation of Wnt canonical signaling in C2C12 cells on SLA surfaces (Figure 2(d), ^*^ *P* < 0.01 versus vehicle treated Polished), in spite of the surge of RhoA activity (Figure 2(c)), thus indicating that cell contractility is required for the increase in canonical Wnt activation observed on rough surfaces and that RhoA control of canonical Wnt activation requires cell tension.

**Figure 3:**
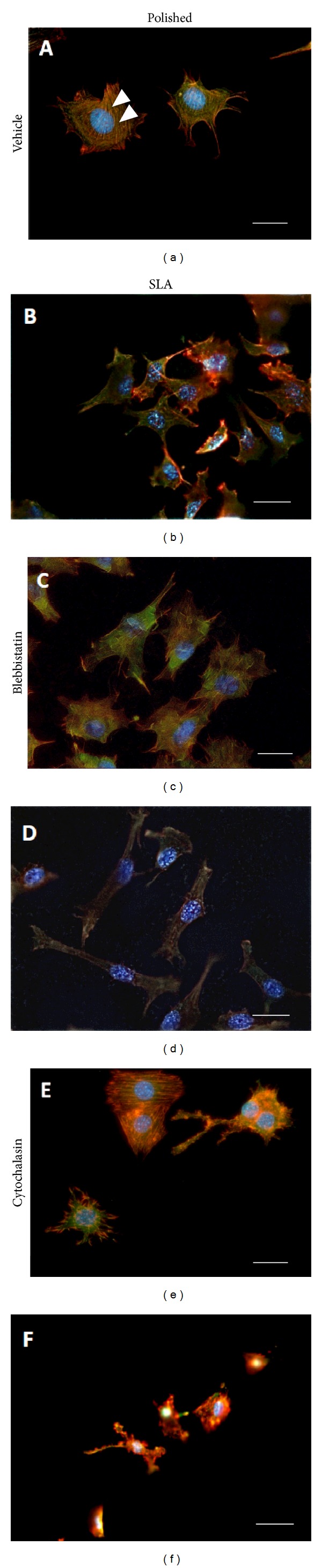
Immunocytochemistry microphotographs (40x magnification, bar 10 mm) of MC3T3 cells growing on Polished (a, c, e) or SLA (b, d, f) titanium discs under basal conditions (a, b), after addition of Blebbistatin (c, d) or Cytochalasin (e, f). Arrowheads indicate stress fibers.

Immunofluorescence microscopy (Figures 3(a)–3(d)) revealed that MC3T3 cells on Polished surfaces had fewer and less evident stress fibers after treatment with Blebbistatin, while cells on rough surfaces appeared more elongated and stretched, presumably for their inability to retract their rear end during migration across the surface.

### 3.4. Cytochalasin Increases RhoA Activation and Affects Cell Differentiation on Titanium

**Figure 4:**
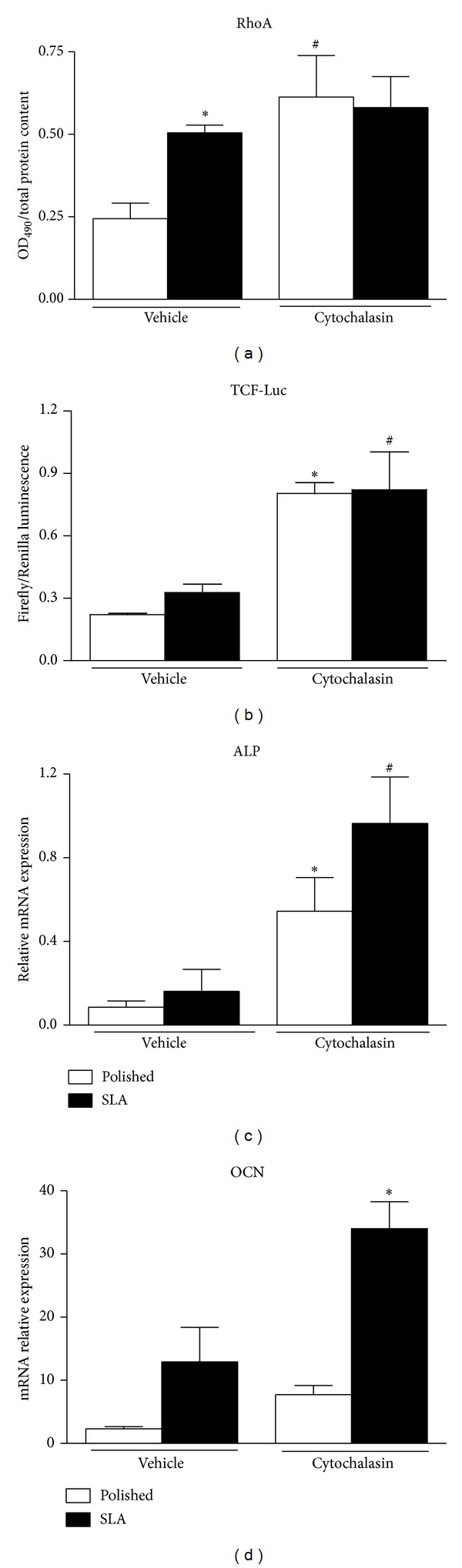
Effects of 0.2 mM Cytochalasin treatment on (a) total RhoA activation levels by G-LISA assay (^*^ *P* < 0.01 versus vehicle-treated polished; ^#^ *P* < 0.05 versus vehicle-treated Polished) in MC3T3 cells and (b) canonical WNT activity by Luciferase reporter assay in C2C12 cells growing on Polished or SLA titanium surfaces. ^*^ *P* < 0.01 versus vehicle-treated Polished. (c, d) Real-time PCR analysis of Alkaline Phosphatase (ALP, ^∗,#^ *P* < 0.01 versus vehicle-treated Polished) and Osteocalcin (OCN, ^*^ *P* < 0.01 versus vehicle-treated SLA) expression in murine osteoblastic MC3T3 cells on Polished or SLA surfaces after Cytochalasin addition.

We then investigated the effects of microfilament structure on MC3T3 cell responses to micropatterned titanium surfaces. Microfilaments are a dense network of actin fibers that permeate cells and can assemble along the main tension lines to form stress fibers and well visible structures at transmission microscopy (Figure 3(a), arrowheads). These are more evident in cells growing on smooth surfaces, than on rough discs (Figures 3(a) and 3(b)), where actin actually appears just as dots or short rods. Cytochalasin is well known to disrupt actin polymerization, and 0.2 mM Cytochalasin solution profoundly altered the conformation of the actin cytoskeleton on both titanium surfaces, at least in part disrupting stress fibers, which appeared fragmented on both surfaces (Figures 3(e) and 3(f)). Cytochalasin affected cell shape as well, inducing irregular cell morphologies. It has been previously reported that Cytochalasin increases RhoA activation, and this was confirmed in MC3T3 cells on Polished discs (Figure 4(a),  ^*^ *P* < 0.01 versus vehicle-treated Polished, ^#^ *P* < 0.05 versus vehicle-treated Polished). Cytochalasin also significantly increased the activation of canonical Wnt signaling in C2C12 cells in both titanium groups (Figure 4(b) ^*^ *P* < 0.01 versus vehicle-treated POlished, ^#^ *P* < 0.05 versus vehicle treated SLA). When osteoblastic MC3T3 cells were treated with 0.2 mM Cytochalasin in osteogenic medium for 3 days, an increase in the expression of osteoblastic markers Alkaline Phosphatase (^*^ *P* < 0.05 versus vehicle treated Polished, ^#^ *P* < 0.05 versus vehicle treated SLA) and Osteocalcin (^*^ *P* < 0.05 versus vehicle treated SLA) was observed (Figures 4(c)-4(d)).

## 4. Discussion

**Figure 5:**
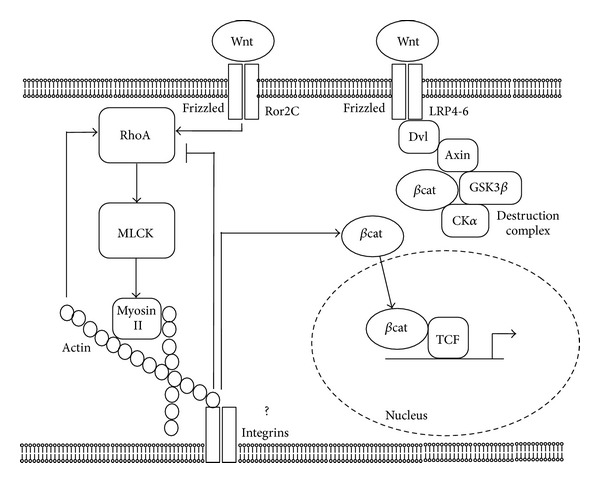
Diagram depicting the proposed model for RhoA role in cell responses to substrate topography. Noncanonical Wnt factors bind to receptor dimers Frizzled-Ror2C, which activate RhoA. Rho, in turns, activates downstream target Myosin Light Chain Kinase (MLCK), which phosphorylates Myosin II. Acto-Myosin filaments create cell tension, which facilitates the activation of canonical Wnt signaling and promotes b catenin release from its destruction complex and translocation to the nucleus possibly by acting on tension sensitive structures (focal adhesions). A feedback loop from tension-activated structures controls RhoA activation levels. Actin microfilaments can control RhoA too, as their partial disruption increases RhoA activity and canonical Wnt activation.

Although there is a large consensus that rough titanium surfaces promote higher expression levels of osteoblastic genes in vitro and bone formation in vivo, the underlying mechanisms are still only partially known. Our group and other research teams have provided evidence that microtextured surfaces can affect the activation of Wnt canonical signaling [5, 7, 13], a pathway required for osteoblastic commitment and differentiation [14, 15]. Understanding how cells can sense the three-dimensional environment that surrounds them and transform geometric cues into intracellular signals could be the key to create tailored implant surfaces to promote bone anabolism and to support bone formation around implanted devices. It is well known that cells can adapt to their culture substrate and conform to its profile: cell shape can correspondently tune to the roughness degree of the surface on which they grow, thanks to an adaptable cytoskeleton. According to Ingber's tensegrity theory, cells reach a state of mechanical balance that maintains their conformation by opposing forces [16, 17]. These are transmitted through rigid struts, formed by cytoskeletal microtubules and tensile wires, the actin microfilaments, anchored to the substrate or the extracellular matrix in a tissue through integrins [18, 19]. This structure is under a proper tension or prestress, provided by Myosin motor proteins that act by pulling on actin filaments [20]. Myosin is activated by a kinase enzyme, known as Myosin Light Chain Kinase, which is controlled, through a signaling cascade, by the small GTPase RhoA, one of the target effectors of the noncanonical Wnt planar cell polarity (PCP) pathway [21]. This signaling cascade plays an important role in orchestrating cell and tissue organization [22] and is triggered by binding of WNT factors, such as WNT5a, to a Frizzled/Orphan tyrosine kinase Ror2 receptor complex [23]. This recruits the Disheveled protein (Dvl) to the cell membrane, activating it [24]. Dvl comprises three molecular domains, which can alternatively trigger the canonical, b-catenin-dependent pathway, and other noncanonical pathways such as the PCP pathway [25–27]. Since cell shape adapts to rough implant surfaces and since the Wnt-related pathways controlling cytoskeletal organization and osteoblastic commitment share some of their key molecular components, we hypothesized that cytoskeletal organization could, at least in part, cause the activation of Wnt signaling observed on rough surfaces. To investigate the relation between cytoskeleton and Wnt signaling activation on implant surfaces, we quantitated active RhoA small GTPase and found that its levels are higher on rough SLA surfaces, according to recent independent data [28]. It can be speculated that cells need to rearrange their cytoskeleton through RhoA activation to adapt to the irregular surface profile of SLA discs. Interestingly, hampering RhoA activity by transfecting cells with a dominant negative isoform of RhoA (dnRhoA) reduced TCF-mediated transcriptional activity on rough surfaces, which we previously showed to be higher than on smooth surfaces under basal conditions [5]. This clearly indicates that RhoA activation is required for the enhancement of Wnt canonical signaling on rough surfaces. However, we also showed that RhoA-mediated canonical Wnt activation required RhoA downstream molecular effectors to be functional. Myosin inhibition by 1 mM Blebbistatin did increase RhoA activity, possibly by a negative feedback loop but failed to enhance Wnt canonical signaling. On the contrary, Wnt activation dropped on SLA surfaces in the presence of Blebbistatin, similar to what observed with dnRhoA. This is consistent with our observation of a complex Myosin distribution in cells growing on SLA surfaces (Figures 2(a) and 2(b)), presumably maintaining adequate cell adhesion by exerting proper cytoskeletal forces on focal adhesions, and it is also consistent with several reports showing that Myosin II function is required for cell differentiation [29, 30]. It can thus be hypothesized that RhoA enhances canonical Wnt signaling by increasing Myosin activation, which in turn is known to activate cytoskeleton-associated signaling molecules such as focal adhesion-associated Kinases (FAKs) [31] (Figure 5). These have been proven to affect the canonical Wnt signaling pathway in certain in vitro and in vivo models [32, 33]. This idea is consistent with recent findings indicating that focal adhesion formation and FAK phosphorylation are controlled by the RhoA pathway that focal adhesion maturation and actin polymerization are promoted in cells cultured on micropatterned substrates [34] and that FAK phosphorylation is required for topography-induced differentiation of human mesenchymal stem cells [35].

In our study, we then treated cells on both surfaces with Cytochalasin. Interestingly, cells growing on Polished surfaces had thick stress fibers and a well-ordered actin network under basal conditions, whilst cells on SLA surfaces, which do not have a plain, uniform surface to adhere on, lacked long actin filaments. It can be speculated that shorter actin filaments can impart a more variegated array of force vectors to the cell membrane and cytoplasm to better adapt to the correspondently irregular titanium surface underneath. Cytochalasin, at this concentration, fragmented the actin cytoskeleton by partially inhibiting its polymerization and significantly affected cell morphology. Cytochalasin also increased RhoA activity, as previously reported, and correspondently increased Wnt activation on both Polished and SLA discs. Under these conditions, cell differentiation markers increased on both surfaces (Figures 4(c) and 4(d)), consistently with reports by Higuchi et al. [36].

Taken together, our results indicate that cells growing on rough surfaces rearrange their cytoskeleton, and this involves the activation of the small GTPase RhoA, which controls downstream effectors that orchestrate cell contractility and shape. Moreover, RhoA can control and is required for the activation of Wnt canonical signaling on rough surfaces. This effect is not exerted directly but through its downstream effector Myosin II. When this is inhibited, Wnt activation on rough surfaces is reversed, in spite of RhoA activation. Our results do not identify the specific mechanism of action for Myosin II, although it may likely include focal adhesion-associated signaling, which is known to be triggered by cell tension and contractility. Actin filaments can control RhoA activity and their fragmentation increases Wnt activation and promotes osteoblast differentiation (Figure 5).

Further studies will be required to better understand how cell contractility allows cells to sense their geometrical microenvironment, but the present study adds another and important piece of evidence to this elusive, yet fundamental puzzle that sustains the integration of osseointegrated implants in bone.
